# Correlation between *GLA* variants and alpha-Galactosidase A profile in dried blood spot: an observational study in Brazilian patients

**DOI:** 10.1186/s13023-019-1274-3

**Published:** 2020-01-29

**Authors:** Patrícia Varela, Gianna Mastroianni Kirsztajn, Fabiana L. Motta, Renan P. Martin, Lauro T. Turaça, Henrique L. F. Ferrer, Caio P. Gomes, Priscila Nicolicht, Maryana Mara Marins, Juliana G. Pessoa, Marion C. Braga, Vânia D’Almeida, Ana Maria Martins, João B. Pesquero

**Affiliations:** 1 Center for Research and Molecular Diagnostic of Genetic Diseases – Department of Biophysics, Federal University of São Paulo, Rua Pedro de Toledo, 669 - 9o andar, São Paulo, 04039-032 Brazil; 2 Division of Nephrology – Department of Medicine, Federal University of São Paulo, São Paulo, SP Brazil; 3 McKeusick-Nathans Institute of Genetic Medicine – Johns Hopkins University School of Medicine, Baltimore, Maryland USA; 4 Department of Psychobiology, Federal University of São Paulo, São Paulo, SP Brazil; 5 Reference Center in Inborn Errors of Metabolism, Pediatrics Department, Federal University of São Paulo, São Paulo, SP Brazil

**Keywords:** Fabry disease, *GLA* gene, Non-coding haplotypes, Molecular diagnosis, α-Galactosidase A activity, DBS enzymatic activity

## Abstract

**Background:**

Fabry disease is a rare X-linked inherited disorder caused by deficiency of α-Galactosidase A. Hundreds of mutations and non-coding haplotypes in the *GLA* gene have been described; however, many are variants of unknown significance, prompting doubts about the diagnosis and treatment. The α-Galactosidase A enzymatic activity in dried blood spot (DBS) samples are widely used for screening purposes; however, even when values below the normal are found, new tests are required to confirm the diagnosis. Here we describe an analysis of *GLA* variants and their correlation with DBS α-Galactosidase A enzymatic activity in a large Brazilian population with Fabry disease symptoms.

**Results:**

We analyzed *GLA* variants by DNA sequencing of 803 male patients with suspected Fabry disease or belonging to high-risk populations; in 179 individuals, 58 different exonic variants were detected. From these, 50 are variants described as pathogenic and eight described as variants of unknown significance. The other individuals presented complex non-coding haplotypes or had no variants. Interestingly, the enzymatic activity in DBS was different among pathogenic variants and the other genotypes, including variants of unknown significance; the first presented mean of 12% of residual activity, while the others presented levels above 70% of the activity found in healthy controls.

**Conclusion:**

The activity of α-Galactosidase A in DBS was markedly reduced in males with known pathogenic variants when compared with subjects presenting variants of unknown significance, non-coding haplotypes, or without variants, indicating a possible non-pathogenic potential of these latter genotypes. These findings bring a better understanding about the biochemical results of α-Galactosidase A in DBS samples, as well as the possible non-pathogenic potential of non-coding haplotypes and variants of unknown significance in *GLA* gene. These results certainly will help clinicians to decide about the treatment of patients carrying variants in the gene causing this rare but life-threatening disease.

## Background

Fabry disease (FD - OMIM 301500) is a lysosomal storage disorder caused by pathogenic variants in the X-linked *GLA* gene (Xq22.1). *GLA* variants may produce α-Galactosidase A deficiency (α-Gal A; EC 3.2.1.22), which is required for degradation of glycosphingolipids. Deficiency in α-Gal A activity leads to storage of complex glycosphingolipids, mainly globotriaosylceramide (Gb3), inside of lysosomes in critical organs and tissues, impairing their functions and consequently resulting in a progressive multisystem disease, affecting people of all ethnic groups [1, 2].

FD presents a broad spectrum of heterogeneous clinical phenotypes, classified as classical and non-classical [3]. In the classical disease, the suspicion of FD begins with peculiar signs and symptoms such as angiokeratomas, acroparesthesia, abdominal pain, recurring headache, and progressive loss of renal function, cardiomyopathy and central nervous system microangiopathy. Non-classical phenotype frequently is associated with damage in a single organ system, principally kidney, heart and brain [4]. The clinical diagnosis of both phenotypes is challenging, since many of the main symptoms are common in other diseases [5]. Indeed, the time between the first symptoms and the diagnosis can take more than ten years.

The FD male phenotypes are directly linked to the residual α-Gal A activity. The exact threshold value of FD pathogenicity is unknown. However, it is estimated that the cutoff for diagnosing FD is 30–35% of mean normal α-Gal A. Some *GLA* mutations cause a reduction of enzyme activity to less than 10–15% of the wild type and are considered pathogenic [6]. However, others promoting a residual enzyme activity of at least 40% of the wild type protein can be considered as non-pathogenic [7].

The enzymatic activity measured in leukocytes or fibroblasts is considered a gold standard for the diagnosis of FD in male patients; however, the sample required for this analysis may be a limiting factor [8]. Thus, the analysis of α-Gal A activity in dried blood spot (DBS) samples has been shown to be a viable alternative, especially for screening in high-risk populations; however, confirmatory tests are required for the diagnosis [9].

The FD molecular analysis is important for family segregation studies, allowing the early diagnosis of family members with pathogenic mutations, enabling the monitoring before the first symptoms and therefore promoting a better management of the symptoms of the disease in these individuals. More than 960 mutations have been reported as causing FD disease in the Human Gene Mutation Database (HGMD) [10]; however, the pathogenicity of several exonic as well as, non-coding variants (NCV) is still controversial. We describe here an observational study based on biochemical analysis performed in DBS samples and genetic analysis in male individuals with suspicion of FD presenting characteristic clinical signs or belonging to high-risk populations, as patients with unexplained renal insufficiency, left ventricular hypertrophy or stroke without a known etiology. The results of the study show the profile of *GLA* variants in male Brazilian patients submitted to investigation of FD and the correlation between α-Gal A activity and genotype.

## Methods

### Patients and α-Gal A activity screening

This study included 803 male patients with suspicion of FD after clinical investigation, as well as individuals with symptoms reported as non-specific, observed in FD patients (high-risk populations). All patients were screened by α-Gal A enzymatic activity, determined by the hydrolysis of the substrate 4-methylumbelliferyl-α-D-galactopyranoside in DBS samples by a fluorometric assay as described by Muller and colleagues [11]. The cut-off value of enzymatic activity (compatible with FD diagnosis) used in this screening protocol was determined in healthy Brazilian volunteers [11] and a pilot screening protocol in DBS samples from Brazilian patients of hemodialysis centers [9].

### DNA sequencing

DNA was extracted from the blood sample using QIAamp DNA Blood Mini Kit (Qiagen, Hilden, Germany), according to manufacturer’s instructions. Alternatively, FTA Classic Cards (Whatman™) were used to facilitate the collection; DNA extraction was performed using Chelex 100 resin (Bio-Rad Laboratories, Hercules, CA, USA) according to instructions. The amplification and sequencing of the *GLA* regions were performed according to Varela and colleagues [12]. We analyzed the seven exons, splicing sites and the regions flanking *GLA* exons. The introns in their entirety, as well as the promoter region, were not sequenced in this study.

### Bioinformatic analysis

Data analysis was carried out using software Geneious® (Biomatters). Sequences were compared with the reference sequence (NCBI: NG_007119 (http://www.ncbi.nih.gov) and confirmed by sequencing the reverse strand. The variants were reviewed and annotated using dbSNP - Single-nucleotide polymorphism database and HGMD – The Human Gene Mutation Database [10]. Based on this analysis, the exonic mutations were divided into described pathogenic variants and variants of unknown significance (VUS). Mutations were correlated with the likely phenotype using the dbFGP - International Fabry Disease Genotype-Phenotype Database [13], previous publications and functional characterization. GnomAD - Genome Aggregation Database [14], 1000 Genomes Project Consortium [15] and ABraOM: Online Archive of Brazilian Mutations [16] were used to define the population frequency. The web-software Human Splicing finder [17] was used to identify significant splicing motif alterations. Non-coding variants were also analyzed by TRAP - Transcript-inferred pathogenicity score [18].

### Non-coding variants analysis

Complex non-coding haplotypes (NCH) were assessed according to their frequency in *The 1000 genomes database*. Briefly, 2504 multisample (phased variant call format - vcf) of the X-chromosome were filtered to rule out variants with two alleles (female sample). The remaining files that comprise 1233 samples of healthy male individuals were used as the control group. A combinatorial analysis was performed to determine the haplotypes. Complex haplotypes found in the 1000 genomes were compared to the patients to determine their frequency.

### Statistical analysis

Correlation between enzyme activity and *GLA* sequencing were analyzed by one-way analyses of variance (ANOVA) with Turkey as *post-hoc*, performed using IBM SPSS® software (version 18). The level of significance was set at *p < 0.05*.

## Results

In this study, we analyzed patients with suspicion of FD with characteristic symptoms of the disease, as well as patients belonging to high-risk populations. Most of the patients presented renal disease and were screened in dialysis clinics (93%), while the other patients presented other symptoms that suggest FD. Details about these data are shown in Additional file 1: Table S1.

All individuals included were screened by α-Gal A enzymatic activity in DBS and presented low activity suggesting a possible FD diagnosis; however, other tests were requested to confirm. Of the total of males submitted to analysis, 783 were screened by enzymatic activity in DBS at the Laboratório de Erros Inatos do Metabolismo (LEIM – UNIFESP), and presented enzyme activity below the cutoff (2.2 μmol/L/h). Other 20 patients included were screened by other laboratories, and the results are reported as positive to FD. These 20 patients presented exonic alterations; therefore, they were included in this study; however, they were not included in the statistical analysis. We performed the *GLA* sequencing to confirm the diagnosis.

*GLA* sequencing revealed 179 patients (22.3%) with mutations in the coding regions (exons), 335 patients had no variants in the analyzed regions (41.7%), and 289 patients (36%) had only NCV. We found 58 previously described variants in the *GLA* exons; 98 patients (12.2%) presented 50 pathogenic mutations and 81 patients (10%) presented eight VUS. The most frequent VUS was D313Y found in 38 index cases, followed by R118C found in 30 individuals. The most frequent pathogenic mutations were R356W and M290I, found in 17 and 10 patients, respectively. A list of described variants, the enzymatic activity, functional tests, and likely phenotype is shown in Table 1.

**Table 1:** Described mutations in the *GLA* gene found in suspected FD patients

Aminoacid change (NM_000169.2)	Nucleotide change	Exon Location	Stop Codon Position	Type of alteration	Nr. of families	Enzymatic activity (μmol/L/h)		dbSNP	HGMD	Functional Characterization (%WT)	Likely Phenotype
						Mean	Range				
Pathogenic Mutations											
p.Q2*	c.4C>T	1	2	Nonsense	1	0.12		rs869312313	DM	No FC	Classical (dbFGP)
p.Gly11AlafsTer110	c.32delG	1	110	Deletion	2	0.09	0 - 0.19	rs1057516967	DM	1.8±1.4 [19]	Classical (dbFGP; HGMD)
p.A15E	c.44C>A	1		Missense	1	0.06		rs869312304	DM	0±0 [42]	Classical (dbFGP)
p.A15G	c.44C>G	1		Missense	1	0.14		No dbSNP ID	DM	19±0.7 [36]	Classical (dbFGP)
p.G35V	c.104G>A	1		Missense	1	0.15		No dbSNP ID	DM	No FC	Classical (HGMD)
p.M42I	c.126G>A	1		Missense	1	0.17		No dbSNP ID	DM	No FC	Likely Classical (dbFGP) / Classical (HGMD)
p.W47*	c.140G>A	1	47	Nonsense	1	+		No dbSNP ID	DM	No FC	Classical (dbFGP)
p.R49C	c.145C>T	1		Missense	1	0.31		No dbSNP ID	DM	0±0 [19]	Classical (dbFGP; HGMD)
p.R49G	c.145C>G	1		Missense	1	0		No dbSNP ID	DM	0±0 [19]	Classical (dbFGP)
p.R49P	c.146G>C	1		Missense	1	0.25		rs398123205	DM	No FC	Classical (dbFGP; HGMD)
p.C52*	c.156C>A	1	52	Nonsense	1	0.41		No dbSNP ID	DM	No FC	Classical (dbFGP; HGMD)
p.F69L	c.207C>A	2		Missense	1	+		No dbSNP ID	DM	No FC	Mild/Late-onset - cardiac variant (dbFGP; HGMD)
p.W81*	c.242G>A	2	81	Nonsense	1	+		rs398123208	DM	No FC	Classical (dbFGP; HGMD)
p.C94Y	c.281G>A	2		Missense	2	0		rs113173389	DM	0±0 [19]	Classical (dbFGP; HGMD)
p.R100K	c.299G>A	2		Missense	1	+		rs869312273	DM	0±0 [20]	Classical (dbFGP; HGMD)
p.R112C	c.334C>T	2		Missense	5	0.25	0 - 1.25	rs104894834	DM	0±0 [20, 42]	Classical (dbFGP; HGMD)
p.R112H	c.335G>A	2		Missense	3	0.17	0.03 - 0.28	rs869312273	DM	0±0 [20]; 1.6±0.6 [42]	Mild proteinuria -Later-onset (dbFGP; HGMD)
p.F113L	c.337T>C	2		Missense	1	0.27		rs869312142	DM	17.3±3.6 [20]; 18.3±0.8 [36]	Later onset (dbFGP); Cardiac variant (HGMD)
p.G132E	c.395G>A	3		Missense	1	0.01		No dbSNP ID	DM	0±0 [42]	Classical (dbFGP)
p.C142R	c.424T>C	3		Missense	2	0	0 - 0.01	No dbSNP ID	DM	0±0 [20, 42]	Classical (dbFGP; HGMD)
p.A156D	c.467C>A	3		Missense	2	0.16	0 - 0.33	rs869312307	DM	0±0 [42]	Classical (dbFGP)
p.C172Y	c.515G>A	3		Missense	1	0.03		rs869312318	DM	0±0 [42]	Classical (dbFGP)
p.M187T	c.560T>C	4		Missense	1	+		rs869312342	DM	0±0 [42]	Classical (dbFGP; HGMD)
p.C202Y	c.605G>A	4		Missense	1	+		rs869312344	DM	0±0 [43]	Classical (dbFGP; HGMD)
p.I198T	c.593T>C	4		Missense	1	0.15		rs727503950	DM	38.7±3.1 [43]	Later onset (dbFGP)
p.W204*	c.611G>A	4	204	Nonsense	1	0.7		rs869312346	DM	No FC	Classical (dbFGP)
p.N215S	c.644A>G	5		Missense	2	0.34	0.22 - 0.47	rs28935197	DM	15.6±1.0 [36] /15.7±2.4 [20] / 39.5±1.5 [43]	Later onset (dbFGP)
p.R220*	c.658C>T	5	220	Nonsense	1	0.06		rs727503949	DM	0±0 [43]	Classical (dbFGP)
p.W226*	c.677G>A	5	226	Nonsense	1	0.48		rs398123219	DM	No FC	Classical (dbFGP)
p.R227*	c.679C>T	5	227	Nonsense	3	0.08	0.06 - 0.11	rs104894841	DM	No FC	Classical (dbFGP; HGMD)
p.R227Q	c.680G>A	5		Missense	1	0		rs104894840	DM	0±0 [20, 42]	Classical (dbFGP; HGMD)
p.Lys240Glufs*9	c.718_719delAA	5	248	Deletion	1	0.01		No dbSNP ID	DM	No FC	Classical (dbFGP)
p.M267I	c.801G>A	5		Missense	1	0.17		rs869312408	DM	No FC	Classical (dbFGP; HGMD)
p.V269M	c.805G>A	6		Missense	1	+		rs869312427	DM	0±0 [43]	Classical dbFGP; HGMD)
p.V269A	c.806T>C	6		Missense	1	0.41		rs28935488	DM	9.0±1.4 [42]	Classical (dbFGP; HGMD)
p.T282I	c.845C>T	6		Missense	1	0.05		No dbSNP ID	DM	5.0±0.5 [42] / 5.2±0.2 [36]	Classical (dbFGP)
p.M290I	c.870G>A	6		Missense	10	0.31	0 - 0.60	rs869312438	DM	39±1.8 [42]	Later onset (dbFGP) - Classical (HGMD)
p.A292V	c.875C>T	6		Missense	1	0.54		No dbSNP ID	DM	No FC	Classical (dbFGP)
p.P293S	c.877C>T	6		Missense	1	0.10		rs869312440	DM	No FC	Classical (dbFGP)
p.R301G	c.901C>G	6		Missense	1	0,34		rs398123224	DM	19±4.1 [42]	VUS (dbFGP)
p.R301*	c.901C>T	6	301	Nonsense	2	0.06	0.01 – 0.11	rs398123224	DM	No FC	Classical (dbFGP) - Kidney disease (HGMD)
p.Gln333Glufs*14	c.996_999delACAG	6	346	Deletion	2	1.84		rs398123229	DM	No FC	Classical (dbFGP; HGMD)
p.R342Q	c.1025G>A	7		Missense	4	0.07	0 -0.14	rs28935493	DM	0±0 [20, 42]	Classical (dbFGP)
p.Ser345Argfs*29	c.1033_1034delTC	7	373	Deletion	2	0.21		rs398123198	DM	No FC	Classical (dbFGP)
p.W349*	c.1046G>A	7	349	Nonsense	1	0,45		No dbSNP ID	DM	No FC	Classical (dbFGP)
p.R356W	c.1066C>T	7		Missense	17	0.47	0.19 - 1.62	rs104894827	DM	16.9±2.3 [42]	Later onset (dbFGP)
p.R363H	c.1088G>A	7		Missense	3	0.43	0.13 - 0.72	rs111422676	DM	31.9±2.9 [42]	Later onset (dbFGP) - Renal presentation (HGMD)
p.Y365*	c.1095T>A	7	365	Nonsense	3	0.06	0 - 0.13	rs104894849	DM	No FC	Classical (dbFGP)
p.W399*	c.1196G>A	7	399	Nonsense	1	+		No dbSNP ID	DM	2% [21]	Classical (dbFGP; HGMD)
p.Thr412Serfs*?	c.1235_1236delCT	7	late termination codon	Deletion	1	0		rs797044777	DM	No FC	Classical (dbFGP; HGMD)
Variants of Unknown Significance											
p.E66Q	c.196G>C	2		Missense	1	+		rs104894833	DFP	52.0±1.3 [36]	Benign (dbFGP)
p.R118C	c.352C>T	2		Missense	30	1.68	1.15 - 2.10	rs148158093	DM	24.0±1.3 [36]	Benign (dbFGP); Cardiac variant (HGMD)
p.A143T	c.427G>A	3		Missense	8	1.04	0.58 - 1.86	rs104894845	DM	31.3±5.6 [42]	Benign (dbFGP)
p.R220Q	c.659G>A	5		Missense	1	1.17		rs727503949	DM?	104±11.3 [42]	Likely bening (dbFGP)
p.N228S	c.683A>G	5		Missense	1	2.03		rs869312152	DM	59.5±9.8 [43] / 124.5±4.1 [36]	Likely benign (dbFGP)
p.D313Y	c.937G>T	6		Missense	38	1.65	0.72 - 2.10	rs28935490	DM?	83.9±21.1 [42]	Benign (dbFGP)
p.R356Q	c.1067G>A	7		Missense	1	0.9		rs869312163	DM	89.1±5.0 [42] / 36.1±1.5 [36]	Later onset (dbFGP)
p.A368T	c.1102G>A	7		Missense	1	1.69	1.69	rs144994244	DM?	103.7±33.6 [42]	Benign (dbFGP)

*F* Female, *M* Male, *No FC* No functional characterization, *NA* Not Applicable, *VUS* Variant of Unknown Significance, *DM* Disease Causing Mutation, *DM?* Disease Causing Mutation?, *DFP* disease-associated polymorphism with supporting functional evidence, dbFGP (International Fabry Disease Genotype-Phenotype Database http://dbfgp.org/dbFgp/fabry/); DBS enzymatic activity applicable only for male: for samples screened by LEIM-UNIFESP the result is show as μmol/L/h; for samples screened by other laboratories, the result is show as + (positive)

### Non-coding variants

Two hundred and eighty nine patients presented only NCV in the *GLA*. A list of all NCV, the population frequency and in silico predictor is shown in Additional file 2: Table S2. Seven NCV form nine NCH. To analyze the frequency of NCH, we used data from *The 1000 Genomes* (only male) as the control group. Except for the haplotype c.-10C > T / c.370-77_370-81delCAGCC / c.640-16A > G / c.802-67G > A / c.1000-22C > T, found only in one patient of this study, all other haplotypes were also found in the control group. The results are shown in Table 2.

**Table 2:** Complex non-coding haplotypes found in male patients with suspicion of FD and population frequency in *The 1000 Genomes* Project

	DNA Region	Variant / Haplotype	Enzyme Activity (μmol/L/h)		Patients		The 1000 Genomes	
			Mean	Range	*N* = 624	%	*N* = 1233	%
	Without *GLA* variants	Without intronic variants	1.76	0.09–2.2	335	53.7	705	57.1
Variant 1	5’UTR	c.-30G > A	1.60	–	1	0.16	2	0.16
Variant 2	5’UTR	c.-10C > T	1.72	1.13–2.10	6	0.74	1	0.08
Variant 3	Intron 1	c.194 + 17A > G	2.11	–	1	0.16	28	2.27
Variant 4	Intron 6	c.1000-22C > T	1.76	0.60–2.19	58	9.29	147	11,92
Haplotype 1	5’UTR / intron 6	c.-12G > A / c.1000-22C > T	1.11	0.80–1.42	2	0.32	1	0.08
Haplotype 2	5’UTR / intron4 / intron 6	c.-12G > A / c.639 + 68 A > G / c.1000-22C > T	1.64	0.90–2.20	23	3.68	127	10.3
Haplotype 3	5’UTR / intron 2 / intron 4	c.-12G > A / c.370-77_370-81delCAGCC / c.640-16A > G	–	–	0	0	1	0.08
Haplotype 4	5’UTR / intron 2	c.-10C > T / c.370-77_370-81delCAGCC	–	–	0	0	1	0.08
Haplotype 5	5’UTR / intron 6	c.-10C > T / c.1000-22C > T	1.81	0.17–2.20	59	9.45	31	2.51
Haplotype 6	5’UTR / intron 4 / intron 6	c.-10C > T / c.640-16A > G / c.1000-22C > T	2.07	–	1	0.16	4	0.32
Haplotype 7	5’UTR / intron 2 / intron 4 / intron 6	c.-10C > T / c.370-77_370-81delCAGCC / c.640-16A > G / c.1000-22C > T	1.72	0.70–2.20	107	17.14	125	10.14
Haplotype 8	5’UTR / intron 2 / intron 4 / intron 5 / intron 6	c.-10C > T / c.370-77_370-81delCAGCC / c.640-16A > G / c.802-67G > A / c.1000-22C > T	2.07	–	1	0.16	0	0
Haplotype 9	intron 2 / intron 4 / intron 6	c.370-77_370-81delCAGCC / c.640-16A > G / c.1000-22C > T	1.68	0.77–2.20	30	4.8	60	4.87

The most frequent haplotype is formed by the four variants c.-10C > T, c.370-77_370-81delCAGCC, c.640-16A > G and c.1000-22C > T. It was found in 107 (17.1%) patients and 125 individuals (10.1%) in the control group. The haplotype formed by c.-10C > T and c.1000-22C > T occurred with a frequency almost fourfold higher in patients with suspected FD than in the control group. The other haplotypes present similar frequency in patients and controls.

### Enzymatic profile

**Fig. 1:**
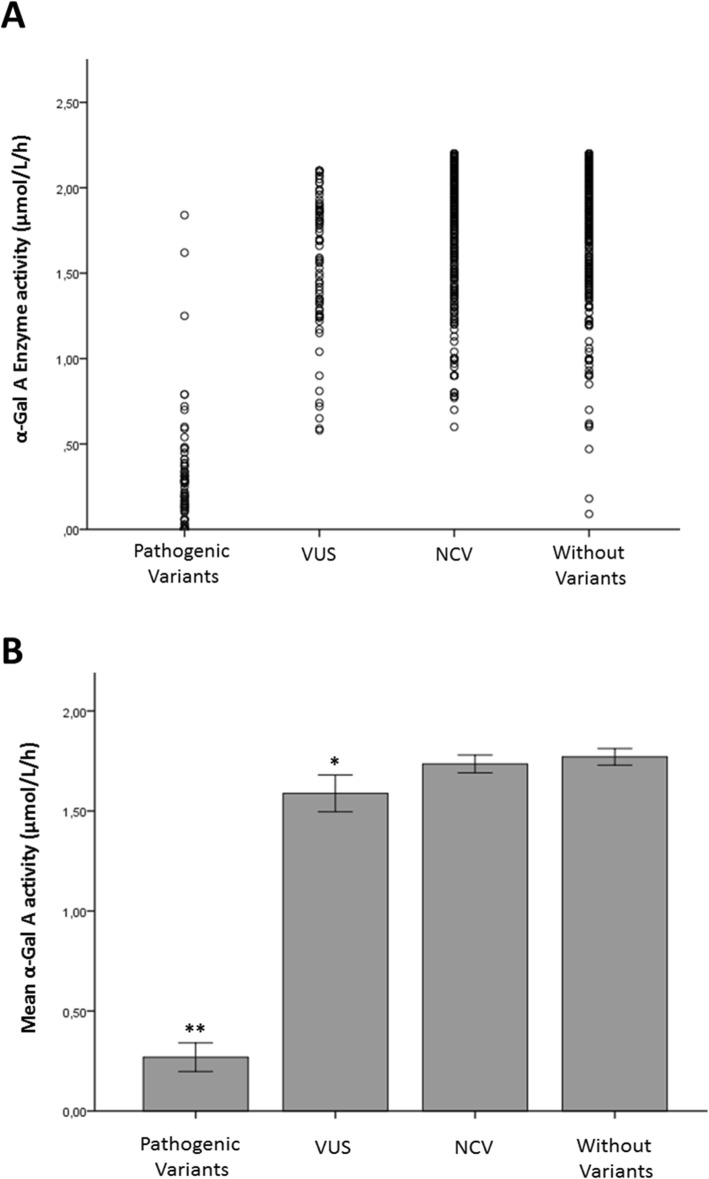
Enzymatic profile of *GLA* genotypes. **(a)** Scatter plot of the α-Gal A activity distribution in males with FD suspicion in different groups. The figure shows that most males with VUS, NCV and without variants present α-Gal A levels above 1 μmol/L/h, while patients with pathogenic variants presented α-Gal A levels lower than 1 μmol/L/h. Some outliers were found in each group. Three patients with pathogenic variants presented enzyme activity above 1 μmol/L/h, while twenty-four patients with only non-coding variants, twenty without variants and seven with VUS, being four with A143T, two with D313Y and one with R356Q, presented enzyme activity below 1 μmol/L/h. **(b)** Correlation analysis between α-Gal A level in DBS and *GLA* genotypes. The graphic shows the mean enzymatic activity detected in males in all the *GLA* variant groups. The data are expressed as mean ± S.E.M. **P < 0.001 known pathogenic mutation (0.27 μmol/L/h ± 0.03, *N* = 83) versus VUS (1.58 μmol/L/h ± 0.04, *N* = 76), non-coding variants (1.73 μmol/L/h ± 0.02, *N* = 289) and the group without variants in *GLA* (1.77 μmol/L/h ± 0.02, *N* = 335); *P = 0.013 VUS versus NCV and *P = 0.01 VUS versus patients without variants

The correlation between the *GLA* variants and the α-Gal A activity levels were evaluated to estimate the impact of the variants in the enzyme in male patients screened by enzymatic activity in LEIM and presenting less than 2.2 μmol/L/h (*N* = 783). The patients were divided into groups according to the classification of their mutations. Figure 1a shows the distribution of enzymatic activity per patient in each group.

Males with variants previously described as pathogenic had significantly lower enzymatic activity when compared with the other groups (mean 0.27 μmol/L/h, *p < 0.001*). The VUS showed a significant decrease of residual α-Gal A levels (mean 1.58 μmol/L/h) when compared with the group without variants (mean 1.73 μmol/L/h; *p = 0.001*) and the NCV group (mean 1.77 μmol/L/h; *p = 0.013*). There was no difference in α-Gal A levels between the group without mutation in *GLA* and the group with NCV (*p = 0.64*). The results are shown in Fig. 1b.

## Discussion

*GLA* sequencing allows the identification of genetic mutations associated with FD and the detection of these variants is fundamental to support the diagnosis. Main FD symptoms are shared with other diseases, making the diagnosis based on such symptoms challenging. The FD clinical suspicion starts with characteristic signs and symptoms that appear over the years, promoting a delay of at least 10 years to diagnose a patient [5]. Therefore, in the last two decades, the number of screening studies in high-risk and in newborn population has increased.

The α-Gal A activity in DBS has been used for screening purposes and should be followed by enzymatic activity in leukocytes or DNA sequencing to confirm the diagnosis [9]. The efficacy of the applicability of enzymatic activity in DBS as an alternative screening test has been reported [8, 22–24]. Fuller and colleagues [25] tested DBS enzyme activity assay in FD hemizygous patients and found a clearly decrease in α-Gal A activity when compared with a control population. A comparison between the enzymatic activity assay in DBS versus leukocytes, conducted in male patients with known FD demonstrated that both assays were equally good [26]. Here, we analyzed by DNA sequencing 803 male individuals with low enzymatic activity in DBS. All patients presented suspicion of FD after clinical investigation or showed undefined symptoms as those observed in FD patients. However, a limitation of this study was the lack of detailed information on the patient’s clinic.

According to Van der Tol and coworkers [27], the prevalence of *GLA* variants in a high-risk population is 0.12%, when considered only pathogenic variants; when VUS are included, this frequency increases to 0.62%. FD is screened in dialysis centers as one possible cause of end-stage renal disease. Not surprisingly, nephrologists referred most patients included in this study, and they were predominantly followed in dialysis services. We performed the DNA sequencing only in individuals with low enzymatic activity screened by DBS assay. Interestingly, we found a high frequency of variants in our patients: 22.2% of individuals with enzymatic activity lower than 2.2 μmol/L/h presented *GLA* variants. Of these, 12.2% present pathogenic variants and 10% VUS. The numbers showed here do not reflect the Van der Tol data, which could be due to the fact that here we included only patients with low activity and not those with activity within the normal range.

In addition to exonic mutations, NCV were also detected. The comparison between patients and controls showed that seven NCV were observed in more than 1% of the control population, being considered as polymorphisms. Other two variants were extremely rare or were not found in any databank consulted. Despite rare, in silico pathogenic analysis did not consider any of the NCV found in this study as damaging.

Nine different NCH were found. Seven of them presented similar frequency in patients and control group. Our results are in agreement with the findings of Ferri and colleagues [28], who found seven different *GLA* haplotypes in control males, indicating that these NCH, per se, are not involved in the development of FD manifestations. However, the haplotypes 5 and 7 present higher frequency in patients when compared with controls. The haplotype 7 was already described in patients with FD suspicion [29, 30]. Both haplotypes contain the variant c.-10C > T, described as causing a decrease of approximately 25% of the α-Gal A activity [31]. As described by Oliveira and colleagues [31], we also found approximately 4-fold higher frequency of this variant in our patients when compared to general population. In our study, these haplotypes were found in males with enzymatic activity below the cut-off (~ 1.73 μmol/L/h), equivalent to a decrease of 21% of α-Gal A activity, indicating that c.-10C > T may cause this decrease. Residual enzyme activity of about 40% of the mean normal level can be considered enough to degrade the substrate, not promoting Gb3 accumulation [6, 7]. However, recent studies have demonstrated that, despite not altering the enzyme structure, patients with the haplotype 7 had significant levels of Gb3 accumulation when compared with controls [32, 33]. Gervas-Arruga and colleagues [32] suggest that in patients with this NCH, environmental factors, as a pro-inflammatory state, in addition to the accumulation of Gb3 may influence the symptoms.

An important finding of this study was the different levels of residual activity in DBS samples among the genotypes. By comparing the mean enzymatic activity, we have observed that described pathogenic variants showed significantly lower mean enzymatic activity, equivalent to 12% of the value found in healthy individuals. On the other hand, VUS including D313Y, R118C, and A143T, considered by many researchers as not causing FD [34–39] and by others as pathogenic [40, 41], presented higher enzymatic activity in comparison to individuals with pathogenic mutations. In contrast, patients carrying VUS present enzymatic levels statistically lower when compared to patients with NCV or patients without mutations in *GLA*. In fact, in in vitro experiments, the VUS found in this study showed a decreased α-Gal A activity, exception for R220Q and A368T, which have α-Gal A activity similar to that of the wild type [36, 42]. However, data from different groups showed that this decrease is not sufficient to promote glycosphingolipid accumulation, which would lead to disease [37, 42]. Although enzymatic activity of VUS were statistically different from NCH and patients without variants, these genotypes present values higher than 70% of the residual activity found in health population. It is already described that activity above 40% of the level found in health population is enough to degrade Gb3, therefore our results indicate that these genotypes are not compatible with FD. However, further studies are necessary to rule out FD in these patients.

In summary, in this study we sequenced a large group of male patients with suspicion of FD presenting enzymatic activity below the cutoff (2.2 μmol/L/h) and showed that pathogenic variants lead to a low residual enzymatic activity, while VUS, NCV and patients without *GLA* variants, lead to approximately 70% of the normal activity, indicating a possible non-pathogenicity. In addition, we showed, by bioinformatics correlation, that the frequency of most haplotypes formed by non-coding variants in the healthy population is similar to the frequency found in patients with suspicion of FD, and therefore, the haplotypes per se, do not correlate with FD. However, in the haplotypes most frequently observed in patients group, although presenting high levels of residual activity when compared with the pathogenic variants, other studies are necessary to discard the FD diagnosis. Moreover, the correlation between DBS enzyme activity and *GLA* variants revealed that this screening method is useful for diagnosing previously described mutations. However, when the patient presents VUS or NCH, although our study indicates a possible non-pathogenicity, the diagnosis may not be conclusive and other tools may be necessary to confirm or discard the disease. Indeed, new specific studies are necessary to correlate these genotypes with FD.

## Conclusions

In this observational study, we identified 98 patients with described pathogenic variants in *GLA* gene, confirming FD diagnosis. In these patients, the enzymatic activity in DBS samples was below 0.3 μmol/L/h, equivalent to 12% of the residual activity of healthy individuals; significantly lower when compared with the other genotypes. On the other hand, 80 patients presented only VUS, and in these cases FD diagnosis was not confirmed, as well as in patients with NCV. Our study indicates a possible non-pathogenic potential of these latter genotypes by population frequency of haplotypes and correlation between the enzymatic phenotype in DBS samples and *GLA* variants. These findings highlight the importance of determining α-Gal A activity by DBS in the diagnosis of FD, considered as the only available tool for this purpose in many countries.

## Supplementary information

**Additional file 1: Table S1.** Origin of samples for FD screening according to the main symptoms

**Additional file 2: Table S2.** Non-coding variants found in *GLA* gene in patients with suspicion of FD and the population frequency in 1000 genomes, GenomAD and ABraOM. Human Splicing Finder and TRAP were used to analyze potential pathogenicity.

## Data Availability

The datasets used and/or analyzed during the current study are available from the corresponding author on reasonable request.

## Abbreviations

ABraOM: Online Archive of Brazilian Mutations
dbFGP: International Fabry Disease Genotype – Phenotype Database
DBS: Dried Blood Spot
FD: Fabry Disease
Gb3: Globotriaosylceramide
GnomAD: Genome Aggregation Database
HGMD: Human Gene Mutation Database
NCH: Non-coding haplotypes
NCV: Non-coding variants
TRAP: Transcript-inferred pathogenicity score
VCF: Variant call format
VUS: Variants of unknown significance
α-GalA: α-Galalactosidase A
