# Supplementary material for: Correlation between GLA variants and alpha-Galactosidase A profile in dried blood spot: an observational study in Brazilian patients
Source: Orphanet J Rare Dis. 2020 Jan 29;15:30. doi: 10.1186/s13023-019-1274-3 (PMC6990533; doi:10.1186/s13023-019-1274-3)
Supplement: Supplementary file 1 — Additional file 1: Table S1. Origin of samples for FD screening according to the main symptoms [file 13023_2019_1274_MOESM1_ESM.docx]

**Table 1S:** Origin of samples for FD screening according to the main symptoms.

|  | Without Variants | Non-coding Variants | **Exonic Variants** | |  |
| --- | --- | --- | --- | --- | --- |
| **Medical Specialities** |  |  | Pathogenic Variants | VUS | **Total** |
| Cardiologist | 2 | 3 | 1 | 0 | 6 |
| General Practitioner | 4 | 6 | 3 | 1 | 14 |
| Dermatologist | 0 | 0 | 1 | 0 | 1 |
| Gatroenterologist | 0 | 2 | 0 | 0 | 2 |
| Geneticist | 5 | 1 | 8 | 1 | 15 |
| Hematologist | 0 | 0 | 3 | 0 | 3 |
| Nephrologist | 318 | 274 | 80 | 78 | 750 |
| Neurologist | 4 | 2 | 2 | 1 | 9 |
| Ophthalmologist | 1 | 1 | 0 | 0 | 2 |
| Rheumatologist | 1 | 0 | 0 | 0 | 1 |
