# Supplementary material for: Correlation between GLA variants and alpha-Galactosidase A profile in dried blood spot: an observational study in Brazilian patients
Source: Orphanet J Rare Dis. 2020 Jan 29;15:30. doi: 10.1186/s13023-019-1274-3 (PMC6990533; doi:10.1186/s13023-019-1274-3)
Supplement: Supplementary file 2 — Additional file 2: Table S2. Non-coding variants found in GLA gene in patients with suspicion of FD and the population frequency in 1000 genomes, GenomAD and ABraOM. Human Splicing Finder and TRAP were used to analyze potential pathogenicity. [file 13023_2019_1274_MOESM2_ESM.docx]

**Table S2:** Non-coding variants found in *GLA* gene in patients with suspicion of FD and the population frequency in 1000 genomes, GenomAD and ABraOM. Human Splicing Finder and TRAP were used to analyze potential pathogenicity.

| **DNA region** | **Variant** | **dbSNP** | **The 1000 Genomes** | | | | | | **GnomAD** | **ABraOM** | **Human Splicing Finder** | **TRAP** |
| --- | --- | --- | --- | --- | --- | --- | --- | --- | --- | --- | --- | --- |
|  |  |  | All | AFR | AMR | EAS | EUR | SAS |  |  |  |  |
| 5'UTR | c.-30G>A | rs3027584 | 0 | 0 | 0 | 0 | 1% | 0 | 0.008 | 0 | NA | 0.207 |
| 5'UTR | c.-12G>A | rs3027585 | 10% | 17% | 12% | 5% | 2% | 13% | 0.060 | 0.08 | NA | 0.002 |
| 5'UTR | c.-10C>T | rs2071225 | 12% | 8% | 6% | 8% | 7% | 34% | 0.090 | 0.06 | NA | 0.017 |
| Intron 1 | c.194+17A>G | rs2071226 | 2% | 0 | 0 | 11% | 0% | 0% | 0.010 | 0.001 | NSSA | 0.022 |
| Intron 2 | c.370-77_370-81delCAGCC | rs5903184 | 16% | 15% | 8% | 8% | 11% | 35% | 0 | 0.10 | NSSA | NA |
| Intron 4 | c.639+68A>G | rs3027589 | 10% | 17% | 12% | 5% | 2% | 12% | 0.060 | 0.08 | NSSA | 0.043 |
| Intron 4 | c.640-16A>G | rs2071397 | 16% | 15% | 8% | 9% | 11% | 37% | 0.139 | 0.12 | NSSA | 0.315 |
| Intron 5 | c.802-67G>A | rs782238586 | 0 | 0 | 0 | 0 | 0 | 0 | 0 | 0 | NSSA | 0.145 |
| Intron 6 | c.1000-22C>T | rs2071228 | 40% | 82% | 25% | 14% | 13% | 49% | 0.247 | 0.29 | NSSA | 0.025 |

**Legend:** NA: Not applicable; AFR: African; AMR: American; EAS: East Asian; EUR: European; SAS: South Asian; GenomAD – Genome Aggregation Database (<http://gnomad.broadinstitute.org/>), The 1000 genomes browser ([internationalgenome.org/1000-genomes-browsers](http://www.internationalgenome.org/1000-genomes-browsers)) and ABraOM: Brazilian genome variants were used to achieve known population frequency data; Human Splicing Finder (<http://umd.be/HSF3/index.html>): NSSA: No significant splicing alteration detected; TRAP: Transcript-inferred pathogenicity score (TRAP - <http://trap-score.org/about.jsp>): TraP score below 0.459 is enriched for benign variants.
